# A multi-omics dissection of the molecular mechanisms underlying water soaking in fresh-cut watermelon

**DOI:** 10.1186/s43897-025-00229-0

**Published:** 2026-02-04

**Authors:** Xu Liu, Jinhua Zuo, Jiejie Tao, Alisdair R. Fernie, Hongwei Wang, Yuanye Jiang, Lili Ma, Xuelian He, Xinyi Feng, Yuelan Liu, Yanyan Zheng, Yunxiang Wang

**Affiliations:** 1 Institute of Agri-food Processing and Nutrition, Beijing Academy of Agriculture and Forestry Sciences, Beijing Key Laboratory of Fruits and Vegetable Storage and Processing, Key Laboratory of Vegetable Postharvest Processing of Ministry of Agriculture and Rural Affairs, State Key Laboratory of Vegetable Biobreeding, Beijing Vegetable Research Center, Beijing Academy of Agriculture and Forestry Science, Beijing, 100097 China; 2 The College of Food Science and Nutritional Engineering, China Agricultural University, Beijing, 100083 China; 3 Max Planck Institute of Molecular Plant Physiology, Potsdam Golm, 14476 Germany

Fresh-cut watermelon is highly susceptible to water-soaking, a physiological disorder characterized by darkened flesh and a translucent appearance of the tissue (Shi et al. 2020). Water soaking is closely associated with cell wall degradation and cell membrane damage, with ethylene and reactive oxygen species (ROS) identified as key contributing factors (Hurr et al. 2013). While previous research on fresh-cut watermelon has primarily focused on optimizing preservation technologies, the molecular mechanisms underlying water soaking remain poorly understood. Therefore, this study aimed to investigate the molecular basis of water soaking in fresh-cut watermelon during cold storage by employing a multi-omics approach, including transcriptomics, DNA methylation analysis, and metabolomics.

During storage, fresh-cut watermelon displayed a progressive increase in water soaking symptoms, accompanied by a reduction in soluble solids content and the accumulation of hydrogen peroxide (H₂O₂) and superoxide anion (O₂⁻•) (Fig. 1A; Fig. S1, S2). We conducted metabolomic analysis to characterize the metabolic changes associated with this disorder (Fig. S3, S4; Table S1-S6). The identified differentially accumulated metabolites (DAMs) were classified into 18 categories and further grouped into six clusters using K-means clustering analysis (Fig. S3; Table S1-S6). The results revealed that most metabolites associated with water soaking were downregulated, including L-glutamic acid, ferulic acid (sodium), glutathione reduced form (GSH), pyrocatechuic acid, and 2,4-dihydroxybenzoic acid, whereas p-coumaryl alcohol was upregulated (Fig. S3). In addition, significant increases in the levels of D-arabinose and pyridoxal phosphate were observed during storage (Fig. S3), suggesting enhanced metabolic activity related to cell wall degradation and ethylene biosynthesis. Collectively, these metabolic alterations collectively indicate a strong association among ROS accumulation, cell wall disassembly, and ethylene production in the development of water soaking in fresh-cut watermelon. To further elucidate the underlying molecular mechanisms, we conducted a transcriptomic analysis to identify key genes involved in this process (Fig. 1B-D; Fig. S5, S6; Table S7, S8). Differentially expressed gene (DEG) analysis comparing WS-0d to subsequent time points (WS-2d, WS-4d, WS-6d, WS-8d) revealed that DEGs were primarily enriched in five pathways: plant hormone signal transduction*,* MAPK signaling pathway*,* plant–pathogen interaction*,* pentose and glucuronate interconversions*, and* cysteine and methionine metabolism (Fig. S5). With increasing storage time, genes involved in ethylene biosynthesis—such as *S-adenosylmethionine synthase 2/3 (ClSAM2/3)*, *1-aminocyclopropane-1-carboxylate synthase 1/2 (ClACS1/2)*, and *1-aminocyclopropane-1-carboxylate oxidase 1 (ClACO1)*—were upregulated. Similarly, key genes involved in ethylene signaling, including *ethylene receptor 2 (ClETR2)*, *ethylene-responsive transcription factor 1 (ClERF1)*, *serine/threonine-protein kinase CTR1 (ClCTR1)*, *EIN3-binding F-box protein 1 (ClEBF1)*, *mitogen-activated protein kinase kinase 4 (ClMKK4)*, and *ethylene insensitive 3 (ClEIN3)*, also exhibited increased expression (Fig. 1C), suggesting a significant role of ethylene in aggravating water soaking. Concurrently, expression levels of ROS-related genes such as *calcium-dependent protein kinase 1/17/32 (ClCPK1/17/32)*, *respiratory burst oxidase homolog protein C (ClRBOHC)*, and *flagellin-sensing 2 (ClFLS2)* were elevated (Fig. 1B), further contributing to the water soaking phenotype. Additionally, genes associated with cell wall degradation—including *pectin methylesterase 7/36/51/61 (ClPME7/36/51/61)*, *pectate lyase 12 (ClPL12)*, and *polygalacturonase (ClPG)*—also showed an upregulation trend (Fig. S6), which would be anticipated to accelerate cell wall breakdown. Taken together, the upregulation of genes involved in ethylene biosynthesis and signaling, ROS production, and cell wall degradation likely drives the progression of water soaking in fresh-cut watermelon during storage.

**Fig. 1:**
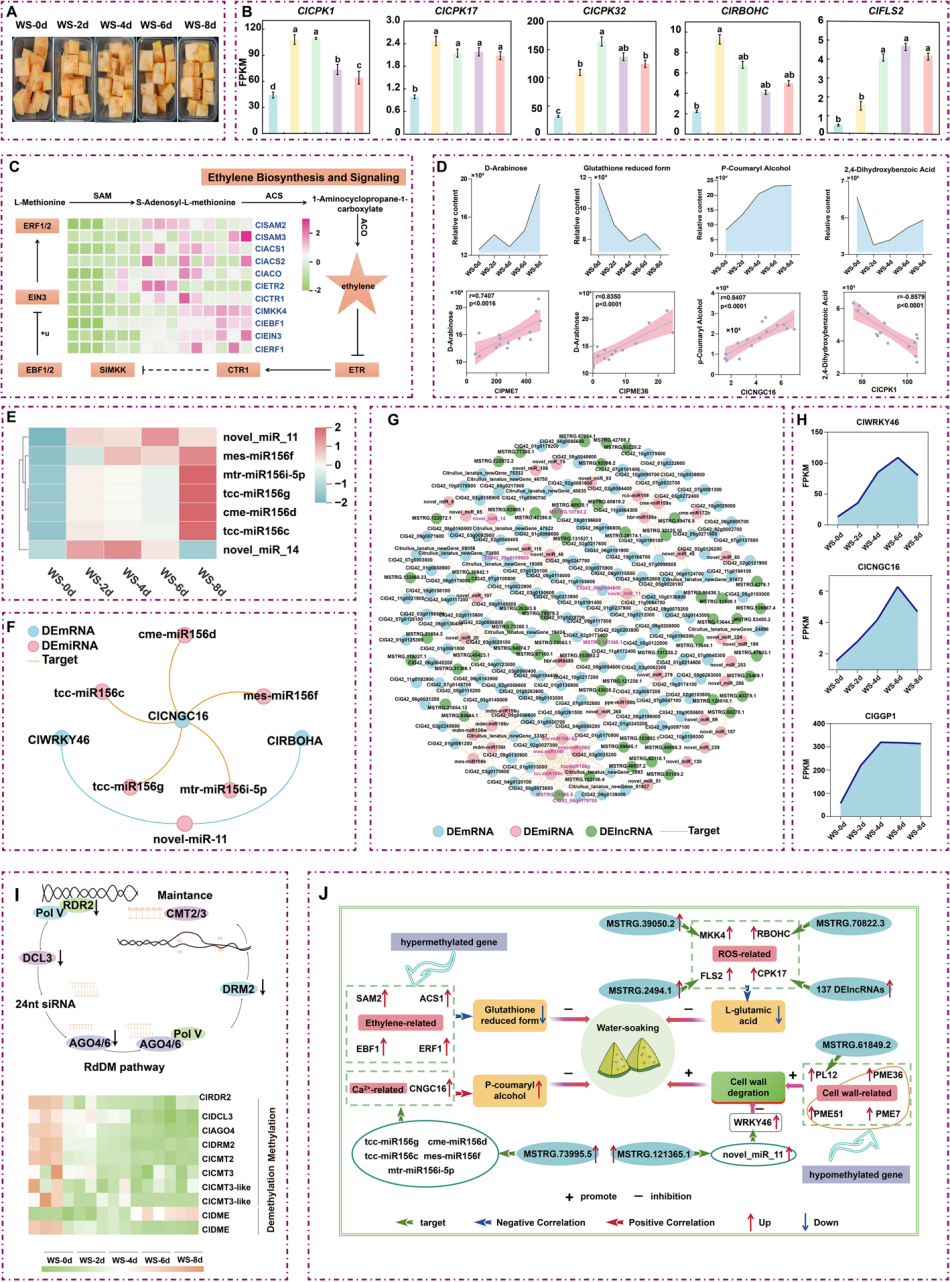
A multi-omics dissection of the molecular mechanisms underlying the response of fresh-cut watermelon to water soaking. **A** Changes in appearance of fresh-cut watermelon during five storage stages. **B** The expression changes of *ClCPK1/17/32*, *ClRBOHC*, and *ClFLS2* genes. **C** Ethylene biosynthesis and signaling pathways. Heat map shows the changes in the expression of genes related to ethylene biosynthesis and signaling. **D** Changes in the content of key metabolites associated with water soaking and correlation analysis between key genes and metabolites. **E** Changes in the expression levels of key miRNAs during storage. **F** Regulatory network of miRNAs and their predicted target genes. **G** Network diagram of lncRNA-miRNA-mRNA. **H** Expression profiles of *ClGGP1*, *ClWRKY46*, *ClCNGC16*, and *ClRBOHA* during storage. **I** The black vertical arrows represent downregulation. The heat map shows the expression changes of representative genes encoding DNA methylases and demethylases during the five storage stages. **J** Proposed model for the water-soaking development mechanism in fresh-cut watermelon. Thick red and blue arrows represent positive and negative correlations, respectively. Thin red and blue arrows indicate gene upregulation and downregulation. Green arrows indicate miRNA targeting mRNA or lncRNA targeting miRNA. Plus and minus signs denote promotion and inhibition, respectively

Genes associated with water-soaking were primarily enriched in the plant hormone signal transduction, MAPK signaling pathway, plant–pathogen interaction, pentose and glucuronate interconversions, and cysteine and methionine metabolism pathways*.* Integrated analysis revealed strong correlations between key DEGs and DAMs, involved in ethylene signaling, ROS production, and cell wall degradation (|Pearson coefficient|> 0.7, *P* < 0.05) (Fig. 1D; Fig. S6), suggesting that they actsynergistically to promote water soaking during storage of fresh-cut watermelon.

To further elucidate the mechanisms of post-transcriptional regulation underlying water soaking, we analyzed non-coding RNAs, including lncRNAs, miRNAs, and circRNAs (Fig. 1E-H; Fig. S7-S9; Tables S9–S13). The target genes of differentially expressed lncRNAs (DElncRNAs) were also predominantly enriched in the plant hormone signal transduction*,* MAPK signaling pathway*,* plant–pathogen interaction*, and* pentose and glucuronate interconversions pathways (Fig. S7). Notably, *ClMKK4*, *ClCPK17*, *ClRBOHC*, *ClFLS2*, and *ClPL12* were all significantly upregulated as targets of DElncRNAs (Fig. S7), thereby promoting the occurrence of water soaking. Interestingly, miRNAs typically bind to the untranslated regions (UTRs) of target mRNAs, suppressing their translation (Fabian et al. 2010). In this study, the novel_miR_11 was predicted to target both *WRKY transcription factor 46 (ClWRKY46) and respiratory burst oxidase homolog protein A (ClRBOHA)*. Additionally, *cyclic nucleotide-gated ion channel 16 (ClCNGC16)* was identified as a target of five different miRNAs (Fig. 1E, F; Fig. S9). *WRKY46* is believed to play a role in maintaining cell wall structure (Bao et al. 2023), *RBOHA* is responsible for ROS generation (Si et al. 2022), and *CNGC16* is involved in calcium signaling (Cui et al. 2020). Changes in the expression of these genes may influence cell wall and membrane integrity, thereby contributing to water soaking in fresh-cut watermelon. For differentially expressed circRNAs (DEcircRNAs), no direct miRNA or mRNA targets involved in the water soaking process were identified (Table S12, S13), this suggesting that circRNAs may not play a significant role in this context. However, previous studies have shown that lncRNAs can contain multiple functional miRNA binding sites and act as "miRNA sponges" to participate in the construction of competing endogenous RNA (ceRNA) networks, thereby indirectly regulating gene expression (Tay et al. 2014). In this study, we constructed a ceRNA network integrating miRNA, mRNA, and lncRNA data. The network included *WRKY46 (ClG42_09g0094600)*, *CNGC16 (ClG42_06g0179700)*, and *GDP-L-galactose phosphorylase 1 (ClGGP1)* (Fig. 1G). *GGP1 (ClG42_09g0109600)* is involved in ROS scavenging (Yang et al. 2024). Notably, all three genes were upregulated despite being targets of miRNAs (Fig. 1H). Their upstream lncRNAs also exhibited high expression levels (Fig. S8). This suggests that the lncRNAs may act as miRNA sponges, competitively binding to miRNAs and thereby reducing their effective concentration. As a result, suppression of the water soaking-related mRNAs was alleviated, enabling their normal or elevated expression. In summary, lncRNAs can directly target water soaking-related genes or act as miRNA sponges to indirectly regulate water soaking-related genes, which in turn affects the watermelon water soaking process.

We also conducted whole-genome bisulfite sequencing (WGBS) on samples collected at different storage stages (Fig. 1I; Fig. S10, S11; Tables S14, S15). Methylation analysis revealed that CG methylation levels in functional regions increased during the first four days and subsequently declined. In CHG and CHH contexts, methylation changes mainly occurred in upstream 2 kb, intron, and downstream 2 kb regions, with the lowest levels at day 0, followed by a slight increase (Fig. S11). Differentially methylated region (DMR) analysis indicated a progressive decrease in hypermethylated DMRs and an increase in hypomethylated DMRs over time, with hyper-DMRs enriched in the CHH context (Fig. S11). Most DMRs were located in promoters and upstream 2 kb regions (Fig. S10). Overall, genome-wide DNA methylation levels declined during storage, especially in the CHH context, consistent with postharvest methylation trends reported in tomato (Li et al. 2024).

Moreover, the expression levels of *chromomethylase 2 (ClCMT2)* and *domains rearranged methylase 1 (ClDRM2)*, which are responsible for maintaining CHH methylation, as well as key genes involved in the RNA-directed DNA methylation (RdDM) pathway, were found to decrease during storage (Fig. 1I). In particular, the downregulation of key RdDM genes such as *ClAGO4* may lead to reduced CHH methylation at multiple RdDM target sites (Duan et al. 2015). Concurrently, the expression of *transcriptional activator DEMETER (ClDME)*, a DNA demethylation-related gene, progressively increased over the storage period (Fig. 1I). These findings suggest that the observed decline in DNA methylation during the progression of water soaking may result from both reduced methylation activity and enhanced demethylation processes.

By integrating transcriptomic and DNA methylation data, we found that the high expression levels of *ClSAM3*, *ClACS*, *ClERF1*, *ClEBF1*, and *ClMKK4* were associated with elevated CHH methylation levels (Fig. S11). In contrast, genes such as *ClACO*, *ClCPK32*, *ClPL12*, and *ClPME7/36/51* exhibited a negative correlation between low CHH methylation and high transcript abundance (Fig. S11). These findings suggest that dynamic DNA methylation changes may be involved in the regulation of water soaking–related gene expression, thereby contributing to the development of water soaking in fresh-cut watermelon.

In summary, water soaking in fresh-cut watermelon is collectively influenced by ethylene, ROS, cell wall degradation-related genes and metabolites, and is further regulated by DNA methylation and non-coding RNAs (Fig. 1J). Our study not only enhances our understanding of the molecular mechanisms underlying water soaking during postharvest storage but also provides a theoretical basis for the precise control of water soaking in fresh-cut watermelon in future applications.

## Supplementary Information

Supplementary Material 1: Table S1. K-means analysis of DAMs. DAMs in cluster I. Table S2. K-means analysis of DAMs. DAMs in cluster II. Table S3. K-means analysis of DAMs. DAMs in cluster III. Table S4. K-means analysis of DAMs. DAMs in cluster IV. Table S5. K-means analysis of DAMs. DAMs in cluster V. Table S6. K-means analysis of DAMs. DAMs in cluster VI. Table S7. Expression of all genes. Table S8. Expression of DEGs. Table S9. Expression of all lncRNAs. Table S10. Expression of all miRNAs. Table S11. Up and down regulation of circRNA. Table S12. Target gene profiles of DEcircRNA in different comparator groups. Table S13. The mRNAs targeted by miRNAs or circRNAs. Table S14. Raw reads were compared with reference genome statistics. Table S15. Number and proportion of hyper- and hypo-DMRs.

Supplementary Material 2: Figure S1. Changes in water-soaking damage ratio and soluble solids content. Figure S2. The changes in the content of superoxide anions and hydrogen peroxide during storage. Figure S3. Metabolomic analysis. Figure S4. KEGG classification map of DAMs. Figure S5. KEGG classification map of DEGs. Figure S6. Transcriptomics analysis. Figure S7. Changes of lncRNA in fresh-cut watermelon during different storage periods. Figure S8. Changes in the levels of key lncRNAs, miRNAs and DEGs. Figure S9. Mapping of miRNA and mRNA binding sites and stem-loop structure figure of novel_miR_11. Figure S10. DNA methylation analysis. Figure S11. Changes of DNA methylation in fresh-cut watermelon at different storage stages. Materials and Methods.

## Data Availability

The datasets used and/or analysed during the current study are available from the corresponding author on reasonable request.
