# Supplementary material for: A multi-omics dissection of the molecular mechanisms underlying water soaking in fresh-cut watermelon
Source: Mol Hortic. 2026 Feb 4;6:20. doi: 10.1186/s43897-025-00229-0 (PMC12870808; doi:10.1186/s43897-025-00229-0)
Supplement: Supplementary file 2 — Supplementary Material 2: Figure S1. Changes in water-soaking damage ratio and soluble solids content. Figure S2. The changes in the content of superoxide anions and hydrogen peroxide during storage. Figure S3. Metabolomic analysis. Figure S4. KEGG classification map of DAMs. Figure S5. KEGG classification map of DEGs. Figure S6. Transcriptomics analysis. Figure S7. Changes of lncRNA in fresh-cut watermelon during different storage periods. Figure S8. Changes in the levels of key lncRNAs, miRNAs and DEGs. Figure S9. Mapping of miRNA and mRNA binding sites and stem-loop structure figure of novel_miR_11. Figure S10. DNA methylation analysis. Figure S11. Changes of DNA methylation in fresh-cut watermelon at different storage stages. Materials and Methods. [file 43897_2025_229_MOESM2_ESM.docx]

**Supplementary Material 1**


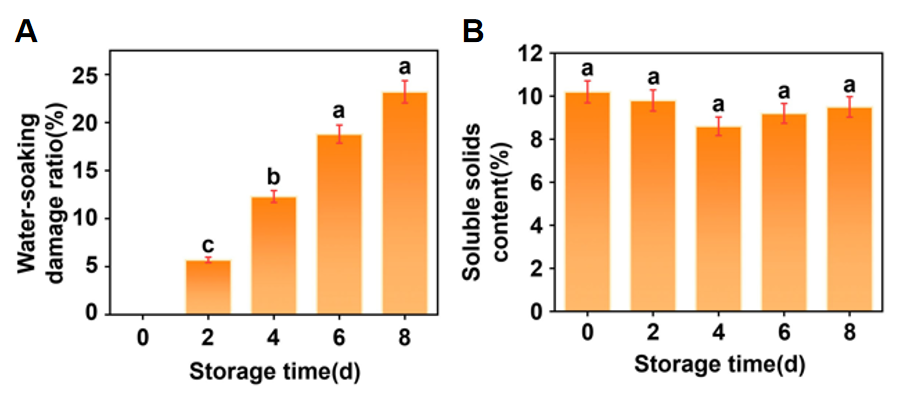


**Fig. S1.** Changes in water-soaking damage ratio and soluble solids content.


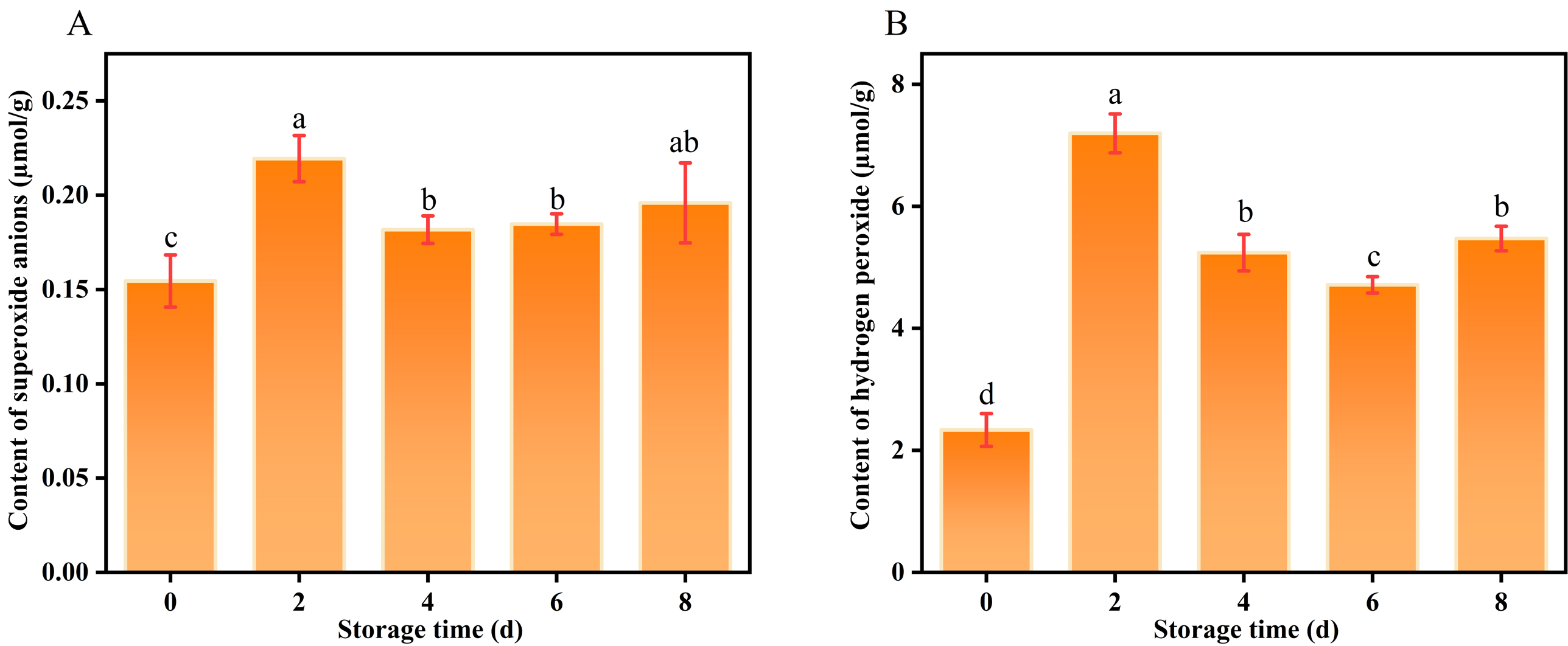


**Fig. S2.** The changes in the content of superoxide anions and hydrogen peroxide during storage.


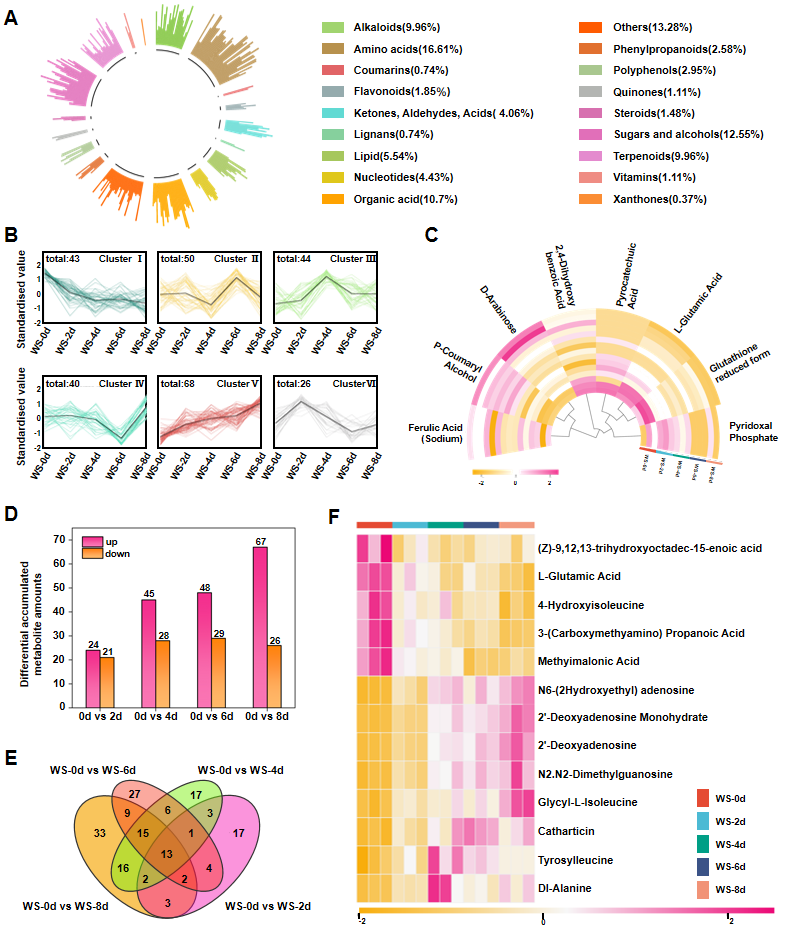


**Fig. S3.** Metabolomic analysis. **(A)** Classification of DAMs. **(B)** K-means analysis of DAMs. **(C)** Key DAMs for Cluster I → VI. **(D)** Statistics of DAMs up and down. **(E)** Venn diagram shows the unique and common DAMs of WS-0d vs WS-2d, WS-0d vs WS-4d, WS-0d vs WS-6d, and WS-0d vs WS-8d. **(F)** Heatmap shows DAMs common to WS-0d vs WS-2d, WS-0d vs WS-4d, WS-0d vs WS-6d, and WS-0d vs WS-8d.


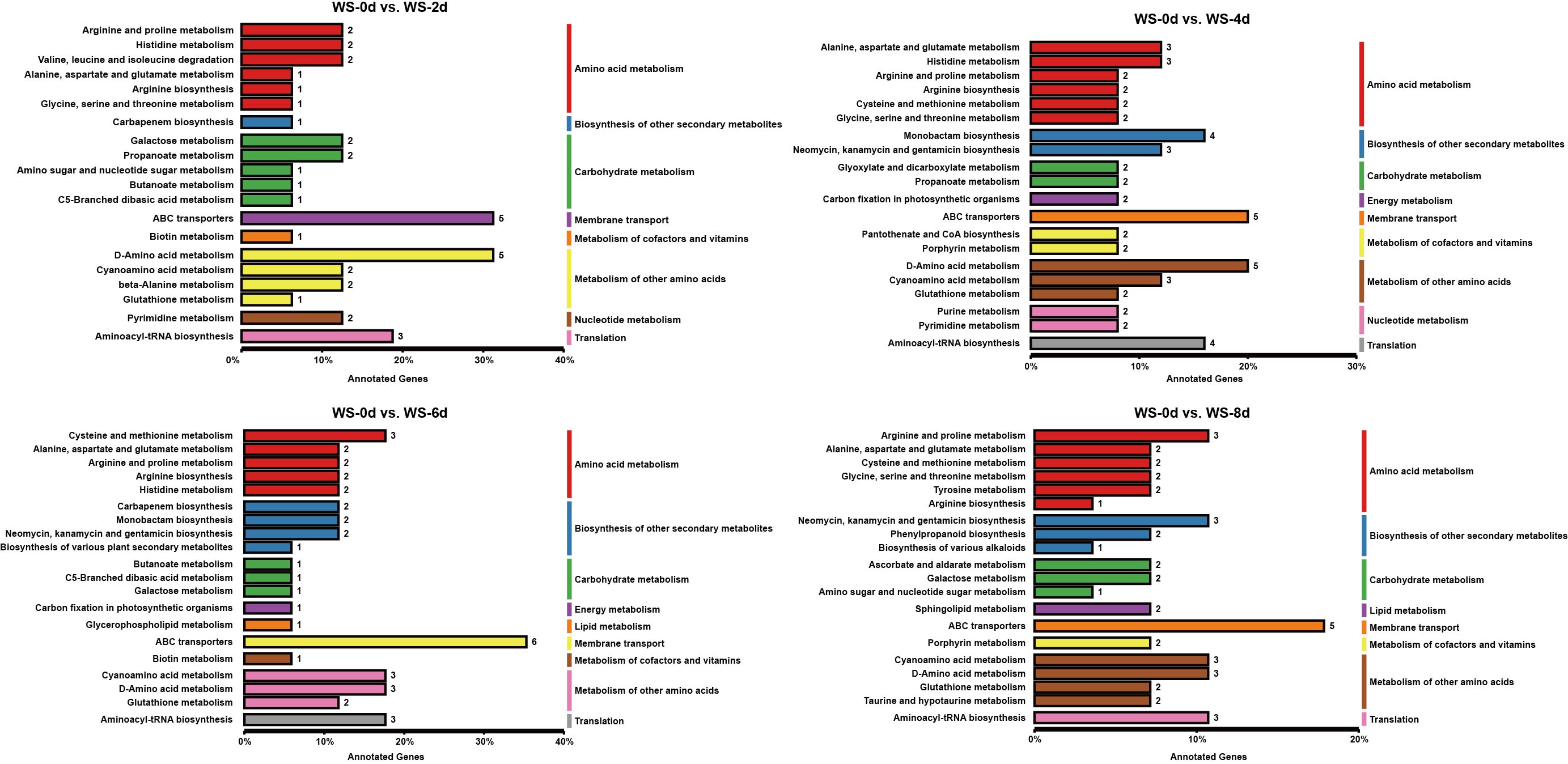


**Fig. S4.** KEGG classification map of DAMs. The ordinate is the name of the KEGG metabolic pathway, and the abscissa is the number of metabolites annotated to the pathway and the proportion of the number to the total number of metabolites annotated.


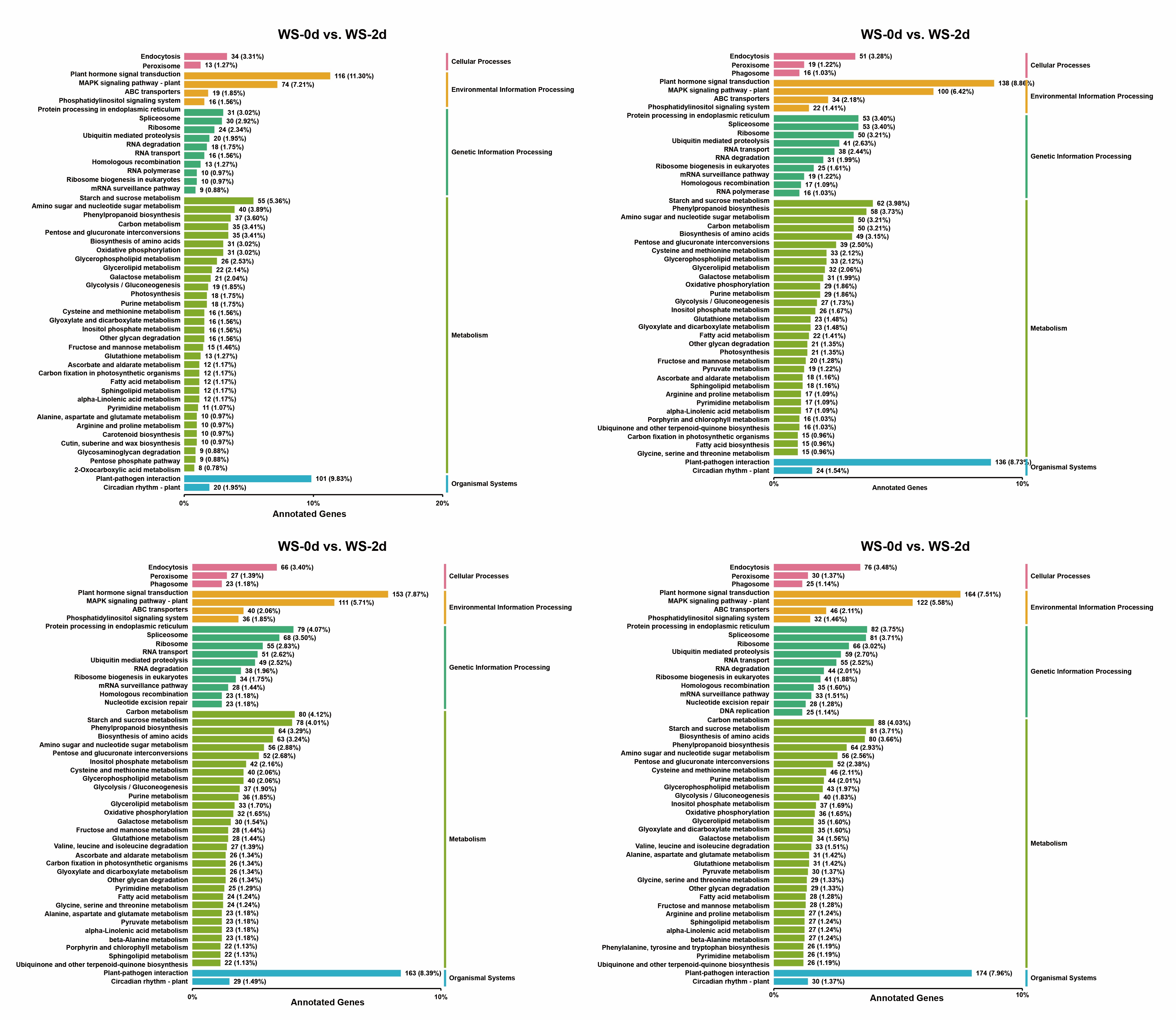


**Fig. S5.** KEGG classification map of DEGs. The ordinate is the name of the KEGG metabolic pathway, and the abscissa is the number of genes annotated to the pathway and the proportion of the number to the total number of genes annotated.


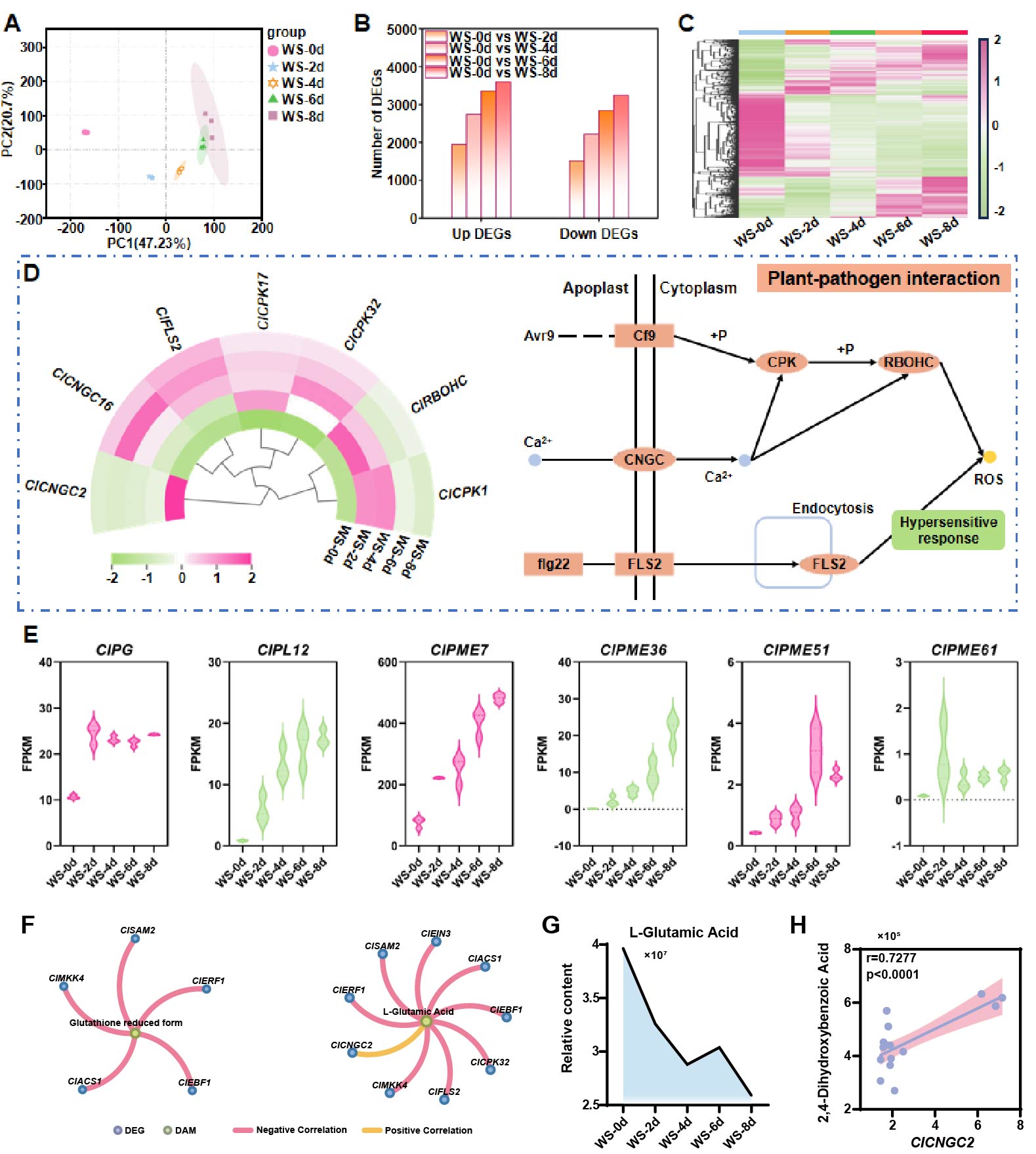


**Fig. S6.** Transcriptomics analysis. **(A) PCA analysis of gene expression. (B) Statistics of up and down-regulated DEGs. (C) Heat map shows the changes in the expression of all DEGs during storage. (D) Plant-pathogen interaction pathway. Heat map shows the expression changes of genes involved in plant-pathogen interaction. (E) Violin plot shows the expression changes of genes involved in cell wall metabolism**. **(F) The correlation network between key genes and metabolites.** (**G**) L-glutamic acid content changes. **(H)** Correlation between 2,4-dihydroxybenzoic acid and *ClCNGC2*.


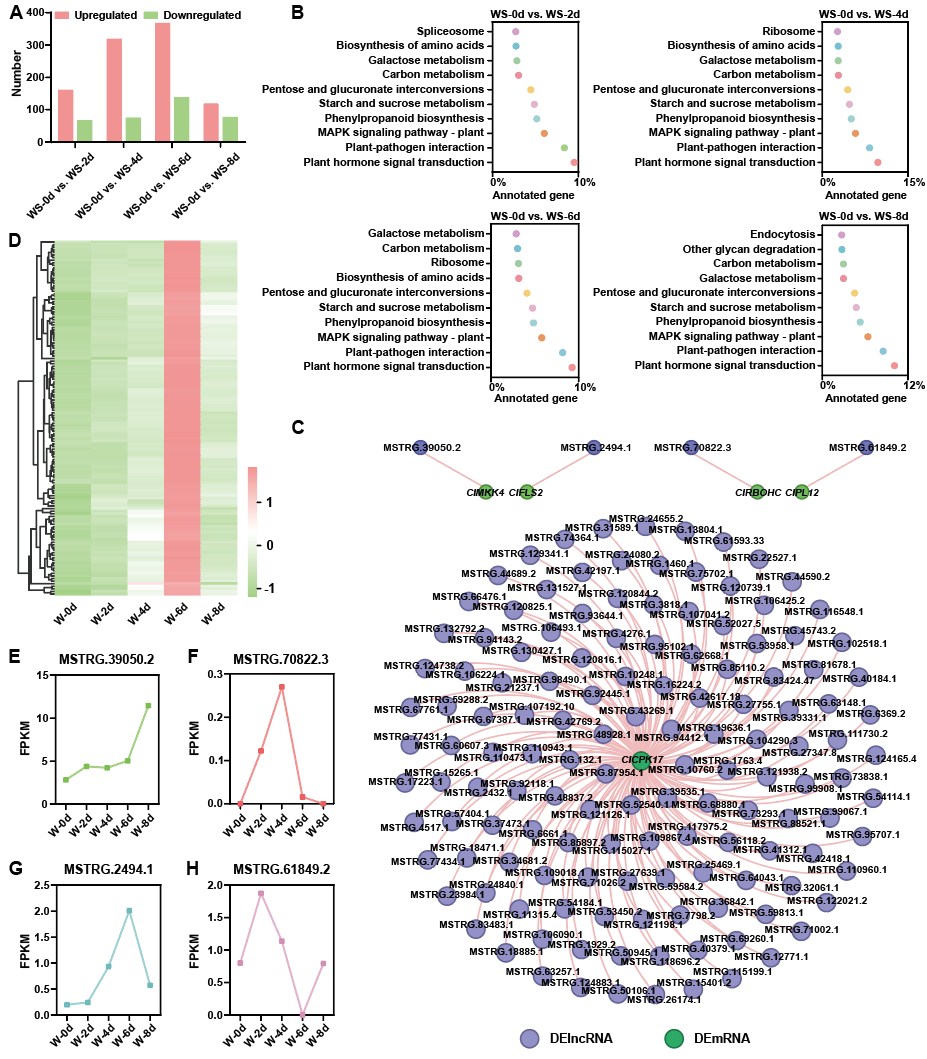


**Fig. S7.** Changes of lncRNA in fresh-cut watermelon during different storage periods. (**A**) Statistics of DElncRNA in different comparison groups. (**B**) KEGG classification map of differentially expressed genes in different comparison groups. The ordinate is the name of the KEGG metabolic pathway, and the abscissa is the ratio of the number of genes annotated to the pathway to the total number of genes annotated. (**C**) Network diagram of the correlation between DElncRNA and its target DEmRNA. (**D**) Heat map shows changes in 137 DElncRNAs targeting *ClCPK17* during storage. (**E**) Changes of MSTRG.39050.2, (**F**) MSTRG.70822.3, (**G**) MSTRG.2494.1, and (**H**) MSTRG.61849.2 during storage.


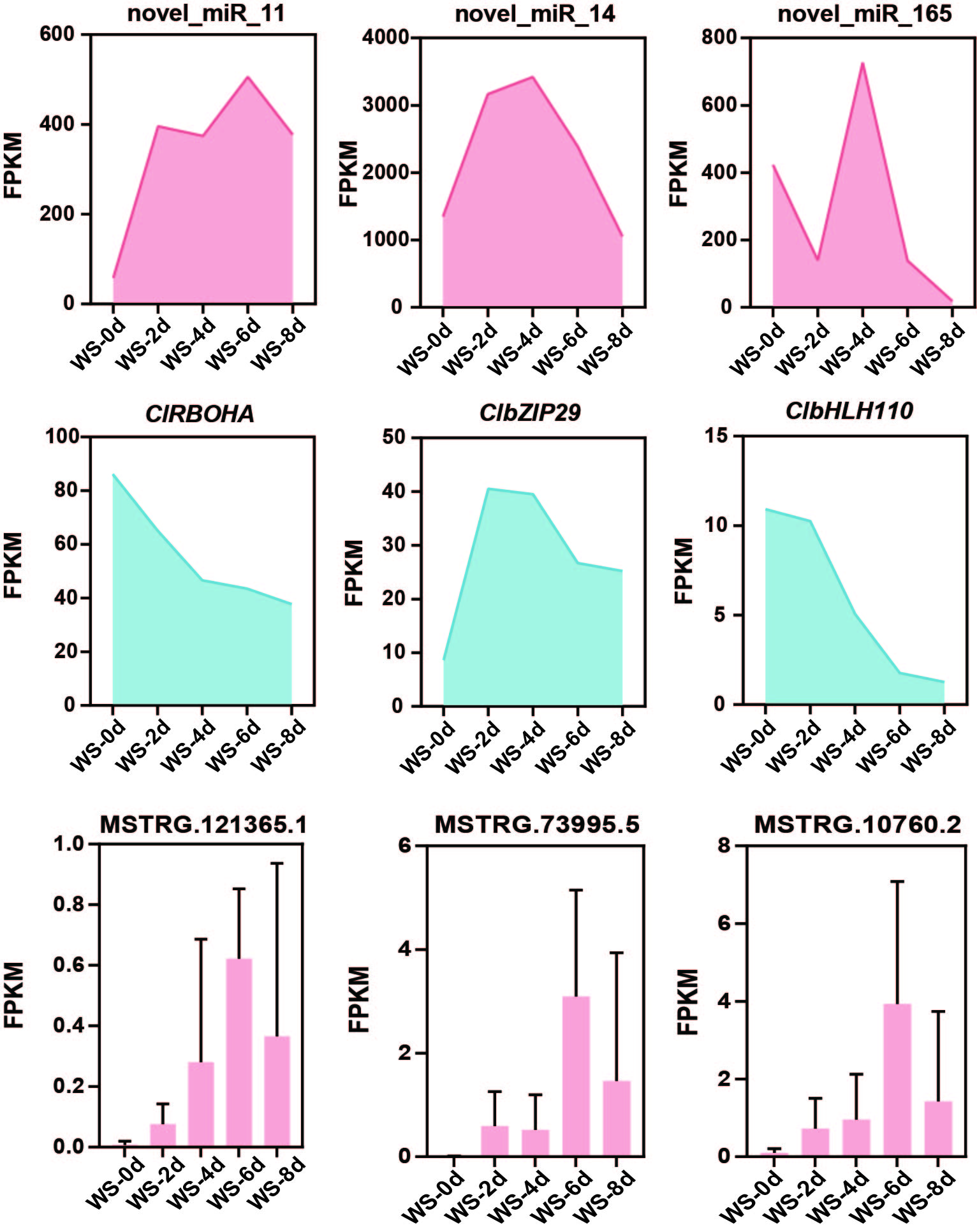


**Fig. S8.** Changes in the levels of key lncRNAs, miRNAs and DEGs.


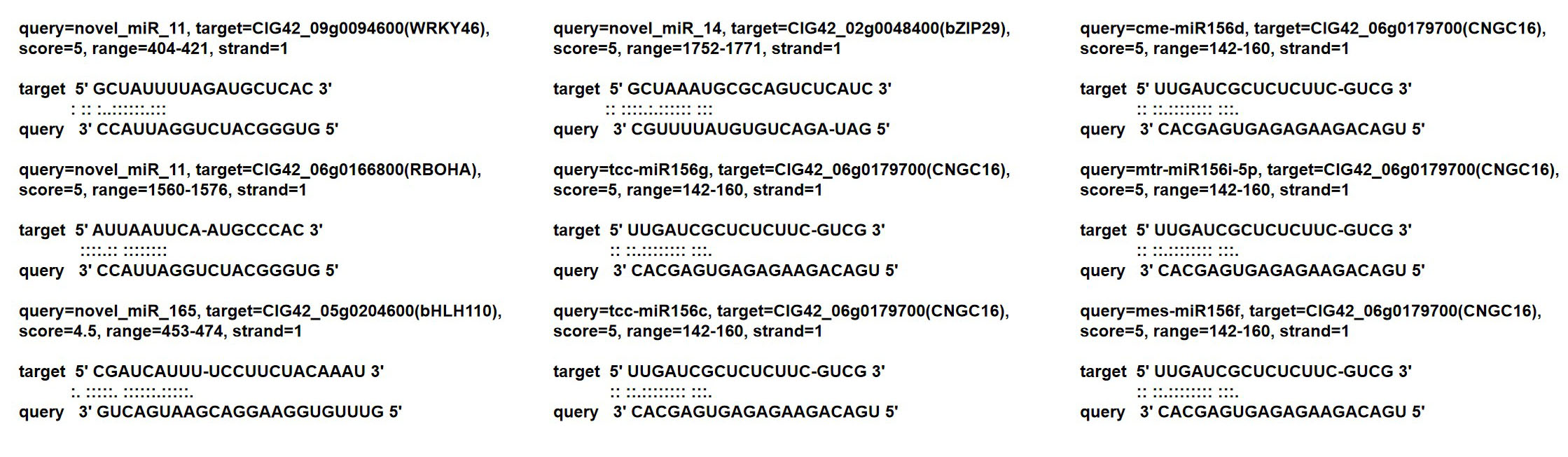


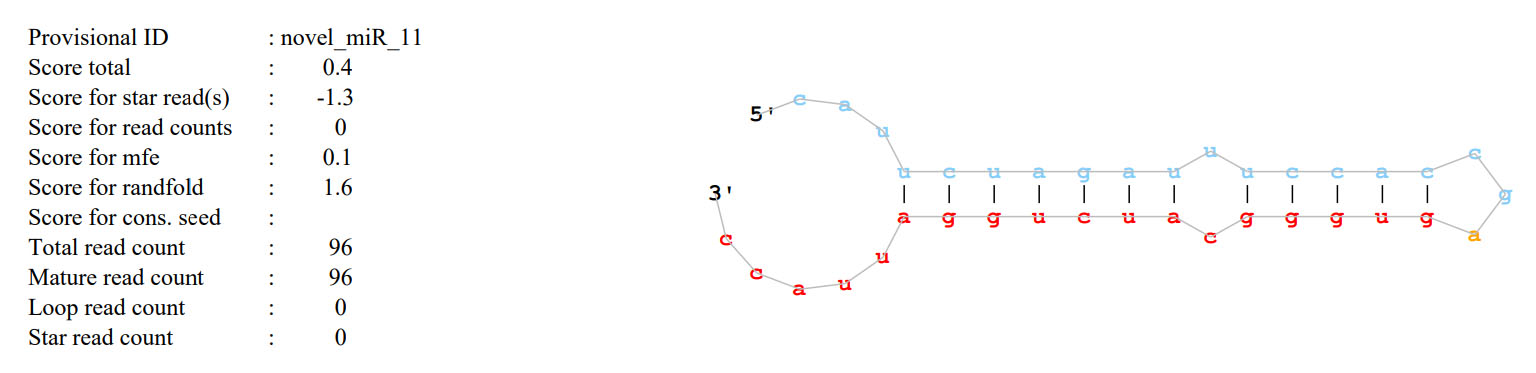


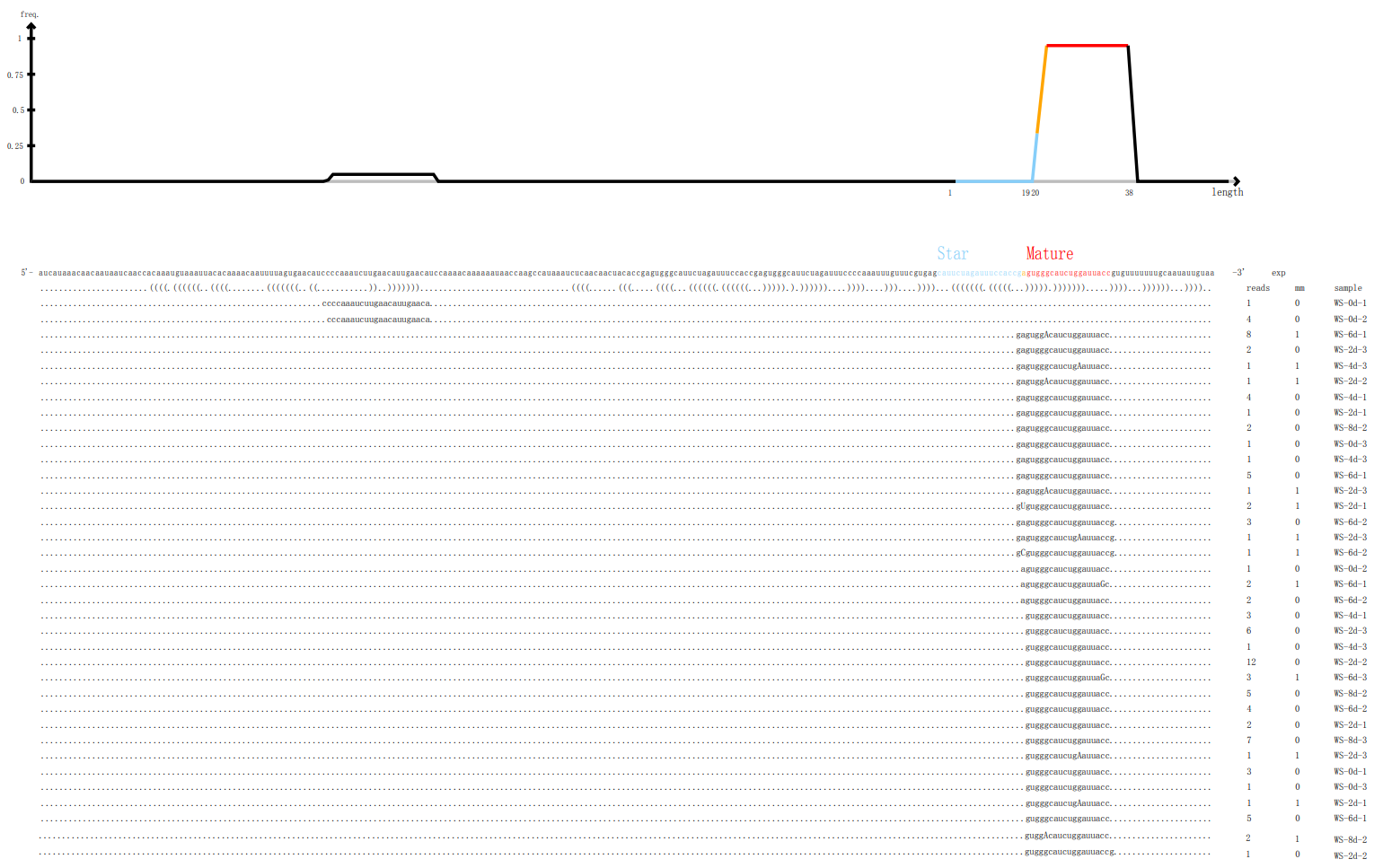


**Fig. S9.** Mapping of miRNA and mRNA binding sites and stem-loop structure figure of novel_miR_11.


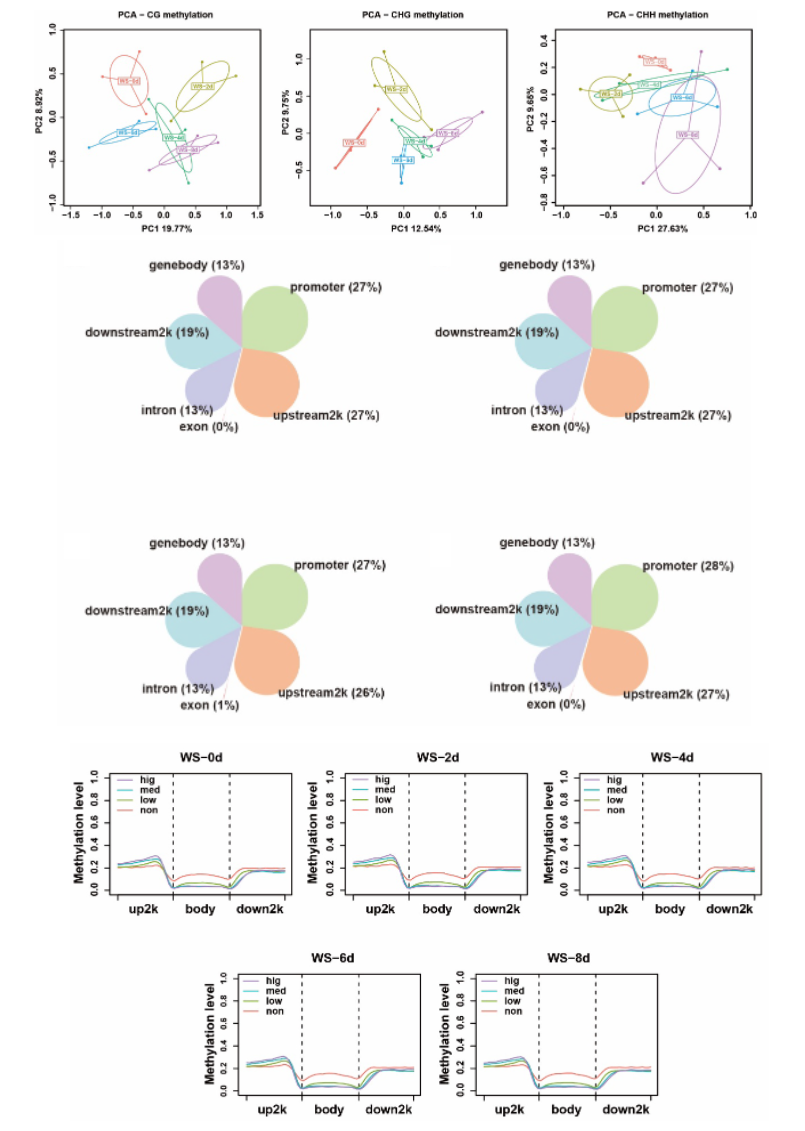


**Fig. S10.** DNA methylation analysis.


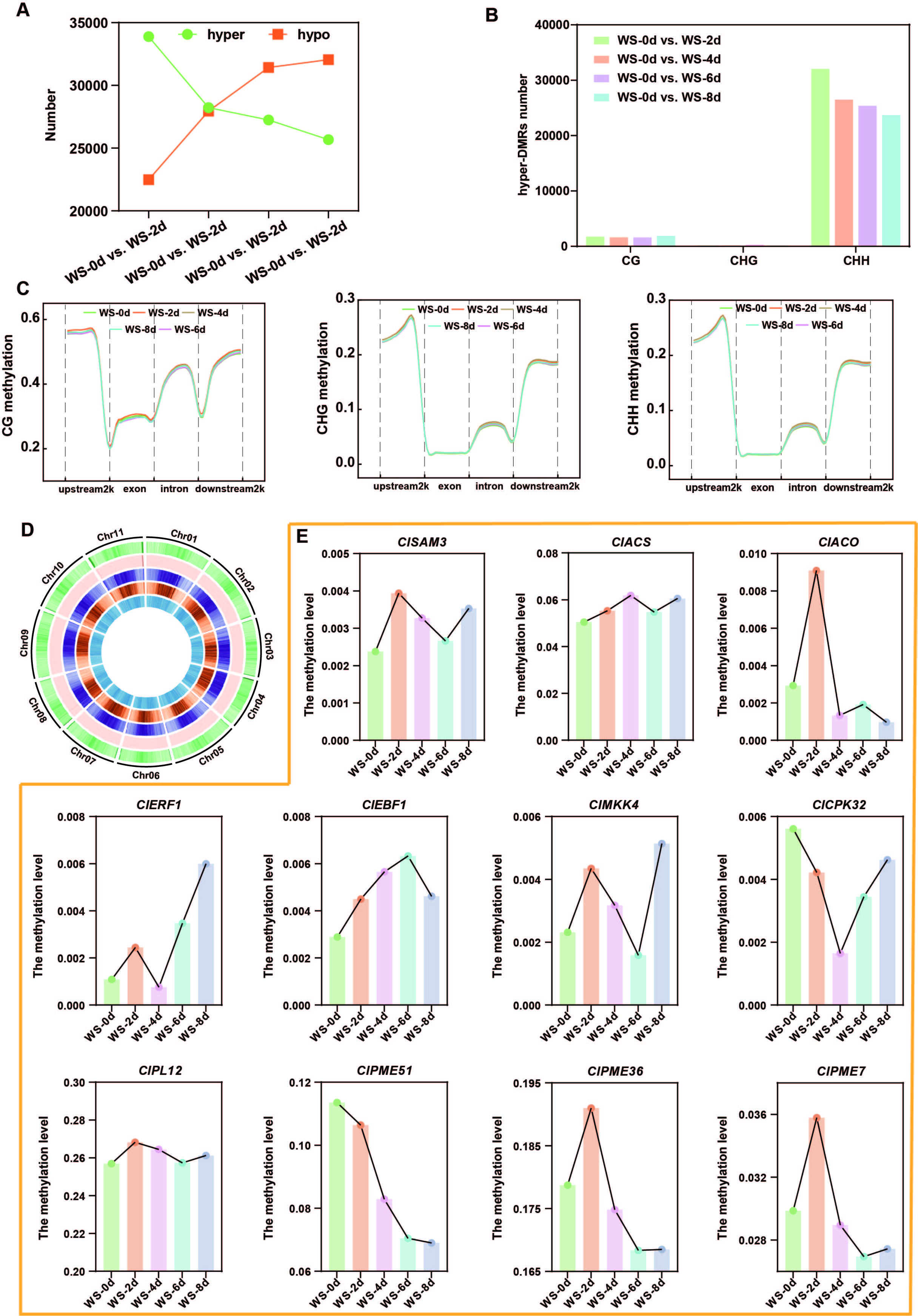


**Fig. S11.** Changes of DNA methylation in fresh-cut watermelon at different storage stages. (**A**) Statistics of hyper-DMRs and hypo-DMRs in each comparison group. (**B**) Statistics of hyper-DMRs and hypo-DMRs in CG, CHG, and CHH backgrounds. (**C**) DNA methylation levels in functional regions in CG, CHG, and CHH backgrounds. (**D**) Circos plots show the distribution of DNA methylation levels on the 11 chromosomes. The six circles from outer to inner indicate the 11 chromosomes, GC content (green), gene density (red), CG methylation level (purple), CHG methylation level (orange) and CHH methylation level (light blue), respectively. The darker the color, the higher the level. (**E**) Changes in DNA methylation levels of *ClSAM3*, *ClACS*, *ClACO*, *ClERF1*, *ClEBF1*, *ClCPK32*, *ClMKK4*, *ClPL12*, *ClPME51*, *ClPME36*, and *ClPME7* at different storage stages.

**Materials and Methods.**

**Sample preparation**

The watermelons were obtained from the “Jingcai” orchard (Yanqing District, Beijing, China). The freshly picked fruits were transported back to the laboratory within one hour, and were immediately pre-cooled at 4 °C for 24 h. The surface of the watermelons was washed with distilled water, and then soaked in 100 mg/L sodium hypochlorite for 2 min to disinfect, rinsed twice with distilled water to remove the disinfectant residue on the surface of the watermelon, and then wiped off the water and dried naturally in a cool place. The watermelons were cut and the inedible parts such as rind were removed, evenly divided into pieces without mechanical damage (about 5 cm³ each), packed into food-grade polyethylene preservation boxes (about 300 g per box), a total of 30 boxes, and stored in a refrigerator at 4 ℃ for 8 days. Three boxes of fresh-cut watermelon fruits were randomly selected on days 0, 2, 4, 6 and 8, respectively, and the water soaking damage and SSC were measured. At the same time, the watermelons were flash frozen in liquid nitrogen and stored at -80 °C for subsequent omics and epigenetic analysis.

**Determination of SSC, water soaking damage ratio and ROS content**

Fresh-cut watermelon fruit pieces were wrapped in gauze (3 randomly selected pieces were placed in the gauze at a time) and the juice was extracted using a pestle and mortar. The SSC of juice was measured using a portable Bailey's saccharimeter (PAL-1, ATAGO, Japan), the assay was repeated nine times.

Three boxes of fresh-cut watermelons were observed and divided into six grades according to the degree of water soaking:0 = no damage (no signs of Water soaking); 1= trace (0% < Water soaking  ≤ 10%); 2 = slight damage (10% < Water soaking  ≤ 20%); 3 = regular damage (20% < Water soaking  ≤ 30%); 4 = moderate damage (30% < Water soaking  ≤ 50%); 5 = severe damage (Water soaking≥ 50%), and the degree of water soaking of each box of fresh-cut watermelon was recorded. The Water soaking index was estimated as follows: Water soaking index (%) = ∑ [(Water soaking level) × (number of fruit at this level)] / (highest level × number of total fruit) × 100 was used to calculate.

The content of superoxide anions and hydrogen peroxide were determined using kits (Beijing Boxbio Science & Technology Co., Ltd, China), and the results were expressed as µmol/g.

**Metabolomics analysis**

Metabolomics analysis was performed on WS-0d, WS-2d, WS-4d, WS-6d, and WS-8d watermelon samples with three biological replicates per sample. Sample preparation, extraction, metabolite identification, and quantification were performed by Biomarker Technologies Co., Ltd. (Beijing, China). The identification of differential accumulated metabolites (DAMs) and KEGG enrichment analysis were performed according to published methods. Kmeans cluster analysis was performed by Metware Cloud, an online data analytics platform (https://cloud.metware.cn). The method iteratively assigns data points to the nearest predefined centroids, updates their positions to minimize intra-cluster distances, thereby partitioning the dataset.

**Sequencing of mRNA, lncRNA, miRNA and circRNA**

mRNA, lncRNA, miRNA and circRNA extraction, library construction and sequencing of watermelon samples were performed by Biomarker Technologies Co., Ltd. (Beijing, China). Raw data after sequencing were processed through the online platform BMCloud (www.biocloud.net) to obtain clean data.

For mRNA, lncRNA, and circRNA, total RNA was extracted using Trizol reagent, rRNA was removed using Epicenter Ribo-Zero, then fragmented and cDNA libraries were constructed for sequencing.

For miRNA, the small RNA Sample Pre Kit was used to create a sRNA library. In short, T4 RNA Ligase 1 and T4 RNA Ligase 2 were used to add the sequencing adapters to the 3′ and 5′ ends of small RNAs. Using the miRNA linked to the adaptor as a template, cDNA was synthesized and a library was constructed for sequencing.

**Identification and analysis of mRNA, lncRNA, miRNA and circRNA**

For mRNA, clean reads were sequence aligned to the reference genome, gene expression was calculated using FPKM, and DEGs were identified under the condition of Fold Change (FC) ≥ 2 and FDR < 0.01.

For circRNA analysis, clean reads from each sample were aligned to the reference genome using BWA (alignment efficiency: 98.60%-99.89%) to generate mapped data. Sequencing library quality control (insert fragment length validation, randomness tests) was performed, followed by circRNA prediction via CIRI. Known and novel circRNAs were classified by database comparison, and Target Finder identified circRNAs that bind to miRNAs. Junction reads were quantified using SRPBM (Spliced Reads Per Billion Mapping) method for expression normalization. DEcircRNAs were screened by DESeq2 with criteria of FC ≥ 2 and FDR < 0.01.

For lncRNA analysis, clean reads were aligned to the reference genome using HISAT2 (Li et al. 2015), followed by transcriptome assembly with StringTie and annotation via gffcompare. Unannotated transcripts were filtered by length (≥ 200 nt) and exon count (≥ 2 exons), then subjected to coding potential assessment using CPC2, CNCI, Pfam, and CPAT to exclude protein-coding candidates. Differential expression analysis was performed with DESeq R package (v1.10.1) based on a negative binomial model, with P-values adjusted by the Benjamini-Hochberg method. FC ≥ 2 and P-value < 0.05 were used as the identification for DElncRNAs, with FPKM as expression quantification metric.

For miRNA analysis, sequence alignment of unannotated reads to the reference genome was performed using Bowtie software. The reads aligned to the reference genome were compared with the mature sequences of known miRNAs and their upstream 2nt and downstream 5nt ranges in the miRBase (v22) database, at most one mismatch was allowed, and the identified reads were considered to be the identified known miRNAs. For the sequences that were not identified to the known miRNAs, the miRDeep2 software obtained the possible precursor sequences through the positional information of the reads compared to the genome, then based on the distribution information of the reads on the precursor sequences and the energy information of the precursor structure (RNAfold randfold). The prediction of new miRNAs was finally realized by scoring the Bayesian model. In the detection of DEmiRNAs, |log2(FC)| ≥ 1 and P-value ≤ 0.05 was used as the screening criterion.

For the functional annotation and enrichment analysis of DEGs, DEcircRNAs, DElncRNAs, and DEmiRNAs, BMKcloud (https://www.biocloud.net/) was used.

**Combined analysis of mRNA and DAMs**

DEGs and DAMs associated with ethylene biosynthesis, ROS metabolism, and cell wall modification pathways were screened and correlated. Pearson correlation coefficients between gene and metabolite expression profiles were calculated using the cor function in R, with significant correlations selected based on an absolute coefficient >0.70 and Pvalue <0.05.

**WGBS and analysis**

Genomic DNA extraction, bisulfite library preparation and sequencing of watermelon samples were performed by Biomarker Technologies Co., Ltd. (Beijing, China) using Illumina HiSeq PE150 platform. Bismark software was used for alignment analysis of methylation data to the reference genome. Methylation site analysis, methylation profile and differential methylation analysis were performed according to the published methods.

**Target gene prediction**

For lncRNA, Perl scripts were used to identify genes within 100 kb upstream and downstream of the lncRNA as cis-target genes of the lncRNA. Pearson correlation coefficient was used to analyze the correlation between lncRNA and mRNA. |r| > 0.9 and P-value < 0.01 were selected as lncRNA trans-target genes.

For miRNA, TargetFinder software was used for target gene prediction based on gene sequence information.

**Statistical analysis**

Data were processed using Microsoft Excel 2021 and plotted using Origin 2024. Statistical significance (P < 0.05) was analyzed using SPSS Statistics 27.0.1, including one-way ANOVA and Duncan's test. The ceRNA network was plotted using Gephi software (v 0.10.1).
